# Complete Genome Characterisation of a Novel 26th Bluetongue Virus Serotype from Kuwait

**DOI:** 10.1371/journal.pone.0026147

**Published:** 2011-10-21

**Authors:** Sushila Maan, Narender S. Maan, Kyriaki Nomikou, Eva Veronesi, Katarzyna Bachanek-Bankowska, Manjunatha N. Belaganahalli, Houssam Attoui, Peter P. C. Mertens

**Affiliations:** Vector-Borne Diseases Programme, Institute for Animal Health, Pirbright, Woking Surrey, United Kingdom; Friedrich-Loeffler-Institut, Germany

## Abstract

*Bluetongue virus* is the “type” species of the genus *Orbivirus,* family *Reoviridae.* Twenty four distinct bluetongue virus (BTV) serotypes have been recognized for decades, any of which is thought to be capable of causing “bluetongue” (BT), an insect-borne disease of ruminants. However, two further BTV serotypes, BTV-25 (Toggenburg orbivirus, from Switzerland) and BTV-26 (from Kuwait) have recently been identified in goats and sheep, respectively. The BTV genome is composed of ten segments of linear dsRNA, encoding 7 virus-structural proteins (VP1 to VP7) and four distinct non-structural (NS) proteins (NS1 to NS4). We report the entire BTV-26 genome sequence (isolate KUW2010/02) and comparisons to other orbiviruses. Highest identity levels were consistently detected with other BTV strains, identifying KUW2010/02 as BTV. The outer-core protein and major BTV serogroup-specific antigen “VP7” showed 98% aa sequence identity with BTV-25, indicating a common ancestry. However, higher level of variation in the nucleotide sequence of Seg-7 (81.2% identity) suggests strong conservation pressures on the protein of these two strains, and that they diverged a long time ago. Comparisons of Seg-2, encoding major outer-capsid component and cell-attachment protein “VP2” identified KUW2010/02 as 26th BTV, within a 12th Seg-2 nucleotype [nucleotype L]. Comparisons of Seg-6, encoding the smaller outer capsid protein VP5, also showed levels of nt/aa variation consistent with identification of KUW2010/02 as BTV-26 (within a 9th Seg-6 nucleotype - nucleotype I). Sequence data for Seg-2 of KUW2010/02 were used to design four sets of oligonucleotide primers for use in BTV-26, type-specific RT-PCR assays. Analyses of other more conserved genome segments placed KUW2010/02 and BTV-25/SWI2008/01 closer to each other than to other “eastern” or “western” BTV strains, but as representatives of two novel and distinct geographic groups (topotypes). Our analyses indicate that all of the BTV genome segments have evolved under strong purifying selection.

## Introduction

*Bluetongue virus* (BTV) is the type-species of the genus *Orbivirus*, the largest of fifteen genera within the family *Reoviridae* [1], [2]. BTV can infect ruminants, camelids, and occasionally large carnivores [3], [4], [5]. The virus is transmitted by biting midges (*Culicoides* spp.) in which it also replicates. It can sometimes also be transmitted either via an oral route, or vertically in sheep and cattle [6], [7]. Clinical signs of BTV infection are often confined to sheep or white-tailed deer and are usually more severe in naïve populations [8], [9]. Cattle and goats are largely (although not exclusively) asymptomatic and can be considered as reservoir hosts [10]. However, the ‘western’ strain of BTV-8 which recently spread across Europe also caused some clinical signs and a low level of mortality in cattle [9].

BTV virus particles are approximately 80 nm in diameter, icosahedral in symmetry and are composed of three concentric protein layers, surrounding a genome composed of 10 linear segments of double-stranded (ds) RNA [11], [12]. BTV genome segments range in size from 3954 to 822 bp (total of 19.2 kbp) and are identified as ‘segment 1 to 10’ (Seg-1 to Seg-10) in order of decreasing molecular weight and/or increasing electrophoretic mobility in 1% agarose gels [1]. Twenty five serotypes of BTV have previously been recognised, the identity of which is determined by the specificity of reactions between neutralising antibodies (generated during infection of the mammalian host) and components of the outer-capsid (VP2 and VP5) [1], [13], [14].

Sequencing studies and phylogenetic comparisons show that Seg-2 and to a lesser extent Seg-6 (encoding outer-capsid proteins VP2 and VP5 respectively) are the most variable components of the BTV genome, varying in a manner that correlates with virus serotype [15], [16], [17]. Sequences of BTV Seg-2 can be divided into 25 distinct clades that correlate exactly with the virus serotype and can be used to identify virus type in sequencing studies or RT-PCR assays. Seg-2 sequences for different serotypes can also grouped into a smaller number of nucleotypes (nucleotypes A to L), which correlate with serological cross-reactions that have been detected between the different BTV types [15], [16], [18].

Structural -proteins, VP3[T2] and VP7[T13] (encoded by Seg-3 and Seg-7) form the innermost ‘sub-core’, and ‘core-surface’ layers of the virus-particle, respectively, and are more highly conserved between BTV serotypes than the outer-capsid proteins [1], [6], [16], [17], [19], [20], [21]. VP7 has been identified as the major *Orbivirus* species / serogroup specific antigen [22] and previous phylogenetic comparisons have used Seg-3 sequences to identify members of individual *Orbivirus* species [23], [24]. BTV also encodes three other highly conserved enzyme-proteins, which represent minor components of the sub-core particle, including: the RNA dependent RNA polymerase - VP1(Pol); the capping enzyme - VP4(CaP); and the helicase VP6(Hel), encoded by Seg-1, Seg-4 and Seg-9 respectively [25].

Four non-structural BTV proteins have also been identified in BTV-infected cells but are not present in purified virions [11], [26], [27], [28]. The two larger and the smallest non-structural proteins (NS1(TuP), NS2(ViP) and NS4) are highly conserved across different BTV serotypes [29], [30]. However, NS3/NS3a can be more variable within some other *Orbivirus* species, representing the second most variable protein of AHSV, after VP2 [31], [32].

The entire BTV genome, including both the ‘conserved’ and more ‘variable’ segments (represented by Seg-2 and Seg-6), show significant nucleotide-sequence variations that at least partially correlate with the geographic origins of the virus isolate / lineage. This suggests that the emergence of individual BTV serotypes was followed by a significant period of geographic isolation allowing mutations to accumulate, generating geographically distinct virus lineages or ‘topotypes’ [15], [16], [17], [33].

Since 1998, multiple BTV types have emerged within Europe, events that have been linked to international trade and climate change in the region, raising concerns about possible future threats posed by bluetongue and other related orbiviral diseases [6], [7], [34], [35]. Multiple exotic BTV types have also been identified (during the same period) in the south-eastern USA [36].

During early 2008, an atypical BTV was detected in clinically healthy goats from the Toggenburg region of north eastern Switzerland, using a BTV-specific real-time RT–PCR (rRT-PCR) targeting Seg-10 designed by Orru et al [37]. Sequence analyses show that this novel strain is distinct from members of the ‘major’ eastern and western BTV topotypes previously identified by Maan et al [16]. Attempts to isolate the virus in cell culture have so far been unsuccessful, making it difficult to confirm its serotype by virus neutralisation tests (VNTs) [38]. However, sera from the infected goats failed to neutralise reference strains of the 24 established BTV serotypes in serum neutralisation tests (SNTs), and together with phylogenetic analyses of Seg-2 nt-sequences, this has identified it as a novel 25^th^ BTV serotype (BTV-25/TOV) [14], [16].

In February 2010, sheep and goats in Kuwait showed clinical signs of disease [39]. Analyses of twenty six blood samples from the Abdali region, identified only two positive samples for BTV, one of which was used to isolate an orbivirus (strain KUW2010/02). VNT using antisera against the existing 25 BTV types failed to neutralise this new virus and it has therefore been proposed as a novel 26^th^ BTV serotype [39]. In order to further characterise the isolate and help determine its relationships, the entire genome of KUW2010/02 was sequenced and compared to other orbiviruses, including multiple BTV isolates. The results from these analyses are presented and discussed.

## Materials and Methods

### Virus isolation, propagation in cell culture

Twenty six EDTA treated blood-samples, five organ-samples (four spleens; one liver) from sheep and goats suspected of infection with BTV, were sent from Kuwait to the OIE reference laboratory for BTV at Institute for Animal Health (IAH) in the UK, during 2010. These samples were taken from naturally infected animals in the field, by qualified veterinarians, as part of normal diagnostic testing procedures in Kuwait and did not therefore require Ethics Committee approval.

Washed blood was inoculated intravenously into embryonated chicken eggs (ECE) (UK Home Office licence number PPL 70/6213), and then passaged twice in BHK-21 clone 13 cells (European Collection of Animal cell Cultures [ECACC – 84100501]) (E1/BHK2). Only one blood sample from Animal No. 374 (which is stored as ‘KUW2010/01’ in the ‘dsRNA virus reference collection’ (dsRNA-VRC) [40] was used successfully to isolate virus (isolate number KUW2010/02). The virus was also passaged twice in Vero cells (ECACC – 84113001) (E1/BHK1/Vero2) until cytopathic effects (CPE) were observed (isolate KUW2010/03).

### Serology

Virus isolates KUW2010/02 and KUW2010/03 were tested by indirect antigen-sandwich ELISA [41] and virus titre was calculated using the Spearman-Karber formula and expressed as TCID_50_/ml.

Virus neutralisation tests (VNT) were performed on KUW2010/02 (using antisera to BTV-1 to BTV-25) to identify the BTV- type in this isolate. A standard ‘constant serum - varying virus’ method was used (with appropriate controls) in a micro titre plates [42]. A ‘neutralisation’ result showing at least 100 fold reduction in virus titre by a ‘type-specific’ reference antiserum, as compared to reactions containing a negative control serum, is regarded as evidence of a specific reaction (same serotype).

### Extraction of RNA and identification of BTV

RNA was extracted from EDTA treated blood using QIAamp Viral RNA Mini Kit (Qiagen) or Universal BioRobot (Qiagen), as per manufacturer's protocol, for use in serogroup and serotype specific real-time RT-PCRs (rRT-PCRs) described earlier [37]. RNA was also purified from infected cells (KUW2010/02 or KUW2010/03) for full-length cDNA synthesis using TRIzol (Invitrogen) [15], [43]. Viral RNA extracted from KUW2010/02 was analysed by agarose gel electrophoresis (AGE) and used for sequencing the entire virus genome.

### RT-PCR for full-length cDNA amplification and sequencing

Full length cDNA copies of BTV genome segments were synthesised and amplified, after ‘anchor spacer–ligation’ as described by Maan et al [44]. ‘Phased primers’ were used to sequence near-terminal regions of Seg-2 and Seg-6, while primers corresponding to conserved 5′ and 3′ terminal sequences were used to sequence the remaining genome segments [16]. The individual cDNA amplicons, purified using a ‘GFX™ PCR DNA and gel band purification kit’ (Amersham Pharmacia Biotech, Inc), were sequenced on a 3730 capillary sequencer (Applied Biosystems). Sequence data for the entire genome of KUW2010/02 have been submitted to GenBank (Table 1).

**Table 1:** Characteristics of dsRNA genome segments (cDNA copy) and proteins of the bluetongue virus serotype 26 Kuwait (KUW2010/02).

Genome segment (Size: bp)	ORFs bp (including stop codon)	Number of Amino acids/Protein nomenclature	Accession numbers	Highest Percentage Identity (nt/aa) with other BTV topotypes				
				Closest Eastern Strain (nt identity)	Closest Eastern strain(s) (aa identity)	Closest Western Strain (nt identity)	Closest Western Strain (aa identity)	BTV-25 (SWI2008/01) [Accession No.] (nt/aa identity)
1 (3944)	12–3920	1302/VP1 (Pol)	JN255156	RSArrrr/16 (BTV-16) (75.3%)	RSArrrr/16 (BTV-16) BUL1999/01 (BTV-9) (87.3%)	RSAvvvv/02 (BTV-2) (75.8%)	NET2007/01 (BTV-8) (87.8%)	[Ac. No. GQ982522] 75.6%/86.8%
2 (2929)	20–2893	957/VP2	HM590642	BTV-23 Australia [Ac. no. U04200] (48.7%)	ISA1991/02 (BTV-23) (38.4%)	BTV-17 USA [Ac. no. AY636073] (62.0%)	BTV-10 USA [Ac. no. U06780] (58.1%)	[Ac. No. EU839840] 63.9%/61.5%
3 (2773)	18–2723	901/VP3 (T2)	HM590643	GRE2001/07 (BTV-1) (75.8%)	RSArrrr/16 (BTV-16) ISR2001/18 (BTV-16) (88.9%)	RSArrrr/09 (BTV-9) (76.4%)	RSArrrr/02 (BTV-2) (88.6%)	[Ac. No. GQ982523] 76.6%/88.9%
4 (1982)	9–1943	644/VP4 (Cap)	JN255157	BTV-12 Taiwan [Ac. no. GU390661] (73.9%)	GRE1999/13 (BTV-16) (81.3%)	BTV-10 USA [Ac. no. Y00421] (74.8%)	USA2003/05 (BTV-5) (81.5%)	[Ac. No. GQ982524] 74.5%/82.3%
5 (1758)	35–1693	552/NS1 (TuP)	JN255158	SER2001/01 (BTV-9) (73.6%)	BUL1999/01 (BTV-9) (80.4%)	NIG1982/07 (BTV-8) (74.4%)	TAT1990/02 (BTV-17) (81.2%)	[Ac. No. EU839841] 72.5%/78.8%
6 (1629)	30–1601	523/VP5	JN255159	AUS1979/02 (BTV-21) (69%)	SER2001/01 (BTV-9) (72.4%)	RSArrrr/24 (BTV-24) (73%)	RSArrrr/11 (BTV-11) (79.3%)	[Ac. No. EU839842] 69.1%/73.4%
7 (1157)	18–1067	349/VP7 (T13)	HM590644	BTV-23 India [Ac. no. AJ277802] (78.6%)	BTV-2 China [Ac. no. AF172826] (94.3%)	BTV-1 France [Ac. no. FJ437558] (79.2%)	USA2003/05 (BTV-5) (94.8%)	[Ac. No. EU839843] 81.2%/97.7%
8 (1121)	19–1080	353/NS2 (ViP)	JN255160	AUS----/01 (BTV-1) (70.8%)	BTV-12 Taiwan [Ac. no. GU390665] (69.4%)	RSArrrr/01 (BTV-1) (71.6%)	TAT1990/02 (BTV-17) (70%)	[Ac. No. EU839844] 69.3%/70.9%
9 (1070)	16–1026 185–415	336/VP6 (Hel) 77/NS4	JN255161	BOS2002/02 (BTV-9) (70.9%)	BOS2002/02 (BTV-9) (64.8%)	BTV-10 USA [Ac. no. U55780] (71.2%)	RSAvvv3/04 (BTV-4) (63.8%)	[Ac. No. EU839845] 73.7%/75%
10 (822)	20–709 59–709	229/NS3 216 NS3a	JN255162	BTV-2 Taiwan [Ac. no. AY493685] (81.3%)	GRE1998/01 (BTV-9) (86%)	BTV-6 South Africa [Ac. no. GQ506497] (80.5%)	NET2007/01 (BTV-8) (86%)	[Ac. No. EU839846] 82.6%/89.5%

### Phylogenetic and positive selection analysis

Consensus sequences for individual genome segments were assembled and analyzed using SeqMan Software (DNAStar Inc.) then aligned with data for other BTV strains from GenBank [16], using CLUSTAL X [45] and MAFFT ver 5 [46].

RevTrans 1.4 Server (http://www.cbs.dtu.dk/services/RevTrans/) [47] was also used for each set of DNA sequences. This translates nt data, aligns the resulting peptide sequences, then uses this ‘scaffold’ to construct multiple DNA alignments that maintain reading frame integrity. A best fit model (selected using the Akaike Information Criterion [AIC] and Bayesian Information Criterion [BIC]) of nucleotide substitution was selected for the coding region of each genome segment [48], for maximum likelihood analysis using Mega 5, as well for positive selection analysis (see below). AIC and BIC selected different nucleotide substitution models for various genome segments of BTV: GTR+G+I (Seg-9); GTR+G (Seg-10); TN93+G+I (Seg-1, -2, -6 and -8); T92+G+I (Seg-3, -4 and -5) and T92+G (Seg-7). Phylogenetic trees from each genome segment were also constructed using neighbour-joining methods and distance matrices, generated by p distance determination algorithm in MEGA version 5 software (500 bootstrap replicates) [48].

The sequence data set for each genome segment was checked for evidence of recombination, using the Genetic Algorithm for Recombination Detection (GARD), www.datamonkey.org/GARD, [49] and Recombination Detection Program (RDP), http://darwin.uvigo.es/rdp/rdp.html [50]. The Tajima D test of neutrality, implemented in MEGA5, was used to assess selection.

For each of these aligned data sets we estimated the rates of non-synonymous and synonymous changes (Positive selection analysis) at each site, using likelihood-based methods as implemented in the on-line Datamonkey server (http://www.datamonkey.org; [49]. These analyses used: i) a conservative single likelihood ancestor-counting (SLAC) method, which is related to that of Suzuki–Gojobori [51] and ii) a fixed-effects likelihood (FEL) method. Both SLAC and FEL methods were used to calculate the global ratio of non-synonymous substitutions per non-synonymous site (dN) to synonymous substitutions per synonymous site (dS) (expressed as dN/dS) using default (estimated) option. A dN/dS ratio ∼1 signifies neutral evolution; dN/dS >1 positive/diversifying selection; and dN/dS <1 negative/purifying selection.

### Development of conventional, gel-based BTV-26 specific RT-PCR assays

RNA from KUW2010/02 was tested by conventional and real-time RT-PCR assays using primers directed against Seg-2 of different BTV serotypes (conventional primers – [52]; real-time assays available from Laboratoire Service International [LSI], Lissieu, France). cDNA amplicons from the conventional assays were analysed by AGE.

Four sets of primers targeting Seg-2 of KUW2010/02 (Ac. No. HM590642) were designed after comparison to multiple BTV isolates of different serotypes [15], [16]. Each primer-pair was evaluated using RNA extracted from BTV-26 (KUW2010/02 and KUW2010/03); BTV-25 (SWI2008/01) (Nucleotype K); and BTV-4, 10, 11, 17, 20 and 24 (the most closely related heterologous serotypes - Nucleotype A) [15]. Primer footprints were compared (*in silico*) with Seg-2 sequences from other BTV serotypes, to confirm type specificity.

## Results

Thirty one blood and tissue samples from Kuwait were tested using four different real-time RT-PCR (rRT-PCR) assays designed to detect BTV RNA [39]. All of the samples gave negative results with assays targeting either Seg-1 [53], or Seg-1 and 5 [54]. However, two blood samples (from animals 364 and 374 [KUW2010/01]) were positive for BTV when tested with an assay targeting Seg-9 (Maan et al – in preparation) and Seg-10 (designed by Orru et al [37]. RNA extracted from KUW2010/01 was also tested by ‘type-specific’ rRT-PCRs targeting Seg-2 (LSI), for European BTV serotypes (BTV-1, 2, 4, 6, 8, 9, 11 and 16), with negative results.

Virus was successfully isolated from one blood-sample (KUW2010/01) and grown in BHK cells (isolate KUW2010/02) or Vero cells (isolate KUW2010/03) [39]. KUW2010/02 and KUW2010/03 were both confirmed as BTV using an indirect sandwich ELISA to detect BTV-VP7 [41] with OD_490_ values >0.15 [39]. KUW2010/02 was also tested in virus neutralisation tests (VNT), using reference guinea pig immune-sera against BTV-1 to BTV-24, as well as BTV +ve antiserum from goats previously infected with BTV-25 (SWI2008/01). None of these antisera caused significant levels of neutralisation, indicating that KUW2010/02 does not belong to previously recognised BTV serotypes (BTV-1 to 25) [39].

Viral RNA extracted from KUW2010/02 was analysed by AGE, and as previously reported [39] generated a migration pattern (electropherotype) typical of BTV, or a closely related orbivirus. RNA from KUW2010/02 was also tested by type-specific, real-time RT-PCR assays, targeting Seg-2 of BTV serotypes 1 to 25 (LSI), with uniformly negative results.

### Sequence and phylogenetic analysis of the genome segments of KUW2010/02

Full-length cDNA copies of KUW2010/02 genome segments were synthesised and both strands of each genome segment were analysed so that consensus sequences could be unambiguously determined. All genome segments have the conserved RNA termini (+ve 5′-GUUAAA...........ACUUAC-3′) that are typical of bluetongue virus [55].

**Figure 1:**
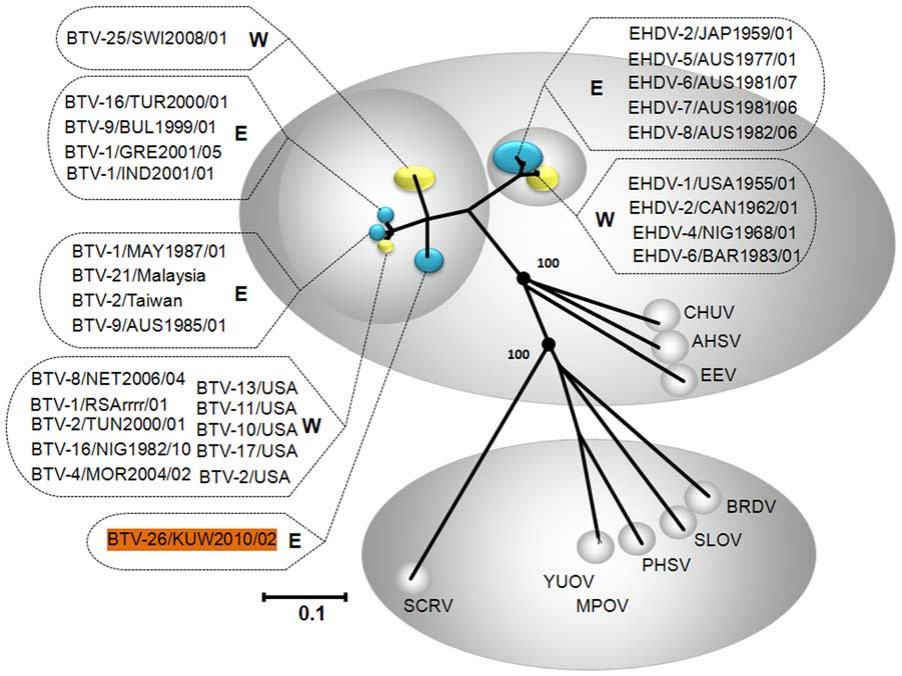
Neighbour-joining tree showing relationships between VP3[T2] of KUW2010/02 with other orbiviruses. KUW2010/02 showed up to 76.6%/88.9% nt/aa identity in Seg-3/VP3[T2] with other BTV strains confirming that it is an isolate of BTV. Accession numbers and further detail of the sequence and viruses used are included in Table 1. The tree was constructed using distance matrices, generated using the p-distance determination algorithm in MEGA 5 (500 bootstrap replicates) [48]. The trees shown in Figures 2 and 3 were drawn using same parameters. The scale bar indicates the number of substitutions per site. Values at the nodes indicate bootstrap confidence. Epizootic haemorrhagic disease virus (EHDV), Bluetongue virus (BTV), Equine encephalosis virus (EEV), African horse sickness virus (AHSV), Chuzan virus (CHUV), St. Croix River virus (SCRV), Yunnan orbivirus (YUOV), Middle point orbivirus (MPOV), Peruvian horsesickness virus (PHSV), Broadhaven virus (BRDV), Stretch Lagoon Orbivirus (SLOV). Eastern and western isolates of EHDV and BTV are shown in blue and yellow respectively. Seg-3 accession numbers used for comparative analyses: AM745079, AM745029, AM745039, AM745049, AM745059, AM744979, AM744999, AM745019, AM745069, NC_005989, AF021236, FJ183386, M87875, NC_012755, NC_007749, NC_007657, EF591620, NC_005998, DQ186827, DQ186797, DQ186822, DQ186811, DQ186816, AF529047, AY493688, DQ186790, AM498052, DQ186792, DQ186826, DQ186819, DQ186817, L19969, L19968, NC 006014, AF017281, L19967.

**Figure 2:**
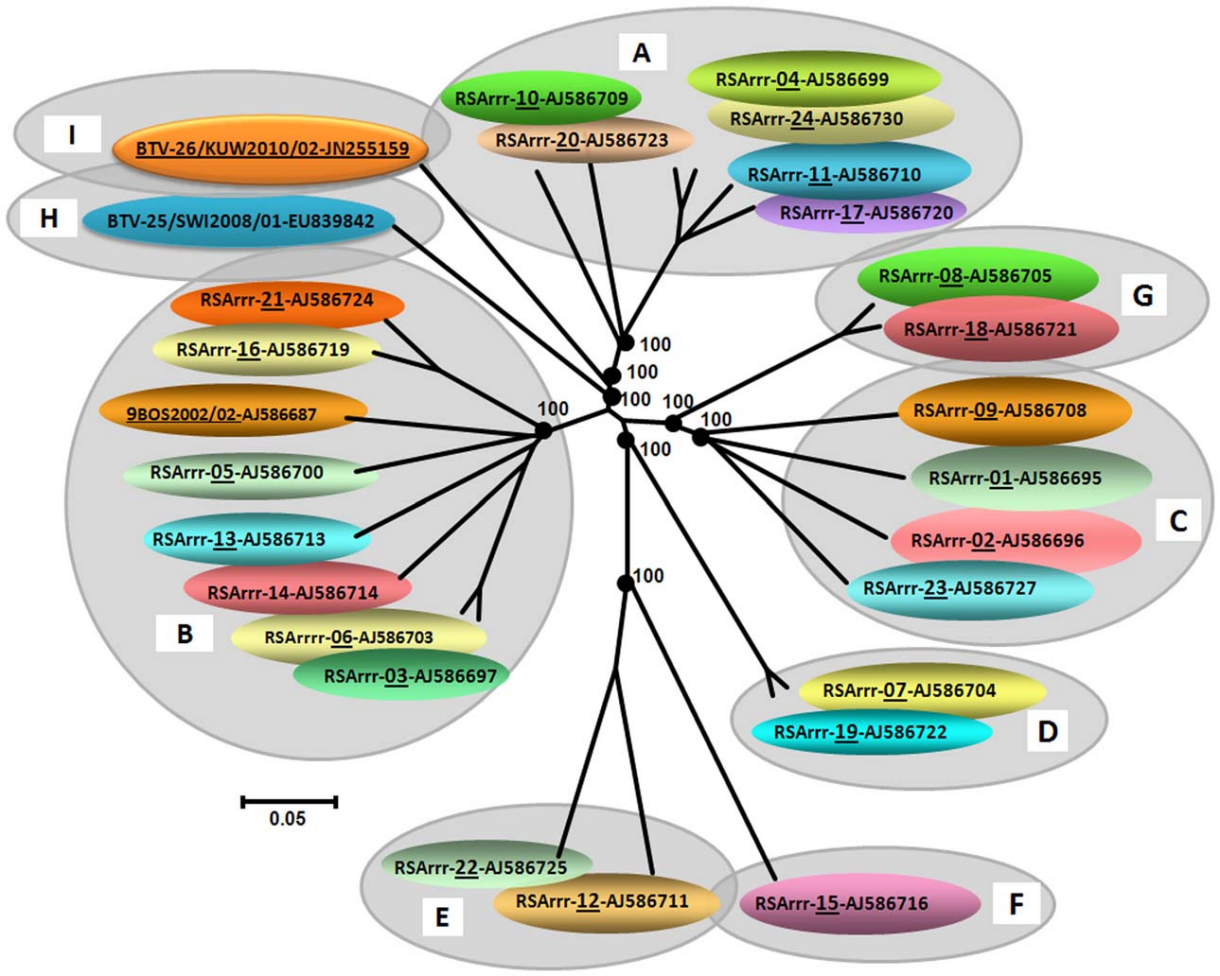
Neighbour-joining tree showing relationships between Seg-6 from KUW2010/02 with the twenty five reference strains of different BTV serotypes. The eight evolutionary branching points are indicated by black dots on the tree (along with their bootstrap values), dividing the sequences into nine ‘Seg-6 nucleotypes’ designated ‘A–I’. In previous studies, eight Seg-6 nucleotypes were identified. Members of the same nucleotype show >76% nt identity in Seg-6, while members of different nucleotypes show <76% nt identity [16]. However the analyses of BTV-26 (KUW2010/02) described here indicate that it forms a new 9^th^ Seg-6 nucleotype (I), as it shows a maximum of 73.0%/79.3% nt/aa identity with previously existing BTV serotypes. Seg-6 accession numbers used for comparative analyses: AJ586695 - AJ586699, AJ586700, AJ586703 - AJ586711, AJ586713, AJ586714, AJ586716, AJ586719, AJ586720 - AJ586725, AJ586727, AJ586730, EU839842.

**Figure 3:**
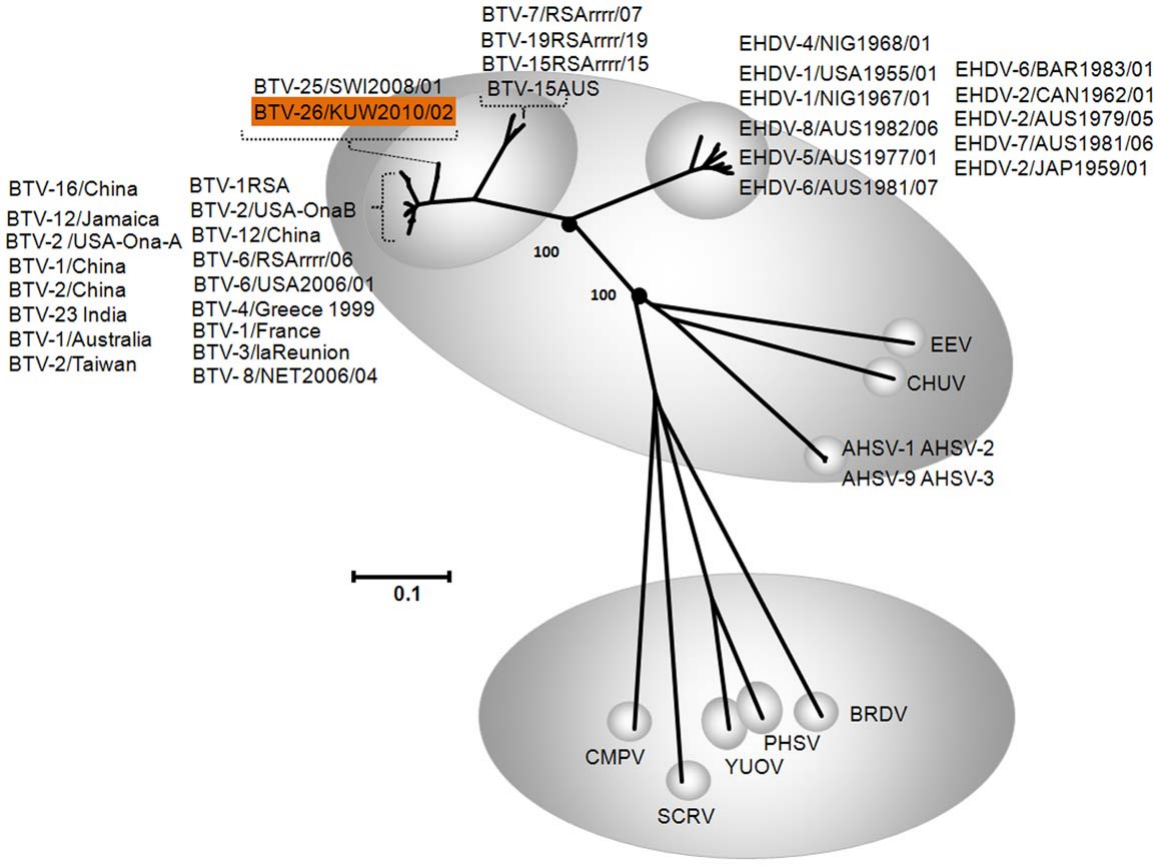
Neighbour-joining tree showing relationships between VP7[T13] from KUW2010/02 with other orbiviruses. KUW2010/02 showed between 69.2%/80.8% to 81.2%/97.7% nt/aa identity in Seg-7/VP7[T13] to other BTV isolates, confirming its identity as a member of the *Bluetongue virus* species. Accession numbers and further detail of the sequence and viruses used are included in Table 1. Epizootic haemorrhagic disease virus (EHDV), Bluetongue virus (BTV), Equine encephalosis virus (EEV), African horse sickness virus (AHSV), Chuzan virus (CHUV), St. Croix River virus (SCRV), Yunnan orbivirus (YUOV), Peruvian horsesickness virus (PHSV), Broadhaven virus (BRDV) and California mosquito pool virus (CMPV). Seg-7 accession numbers used for comparative analyses: AM745023, AM744983, AM745013, AM745063, AM745033, AM745043, AM745073, AM745003, AM744993, AM745053, AM745083, FJ183391, AY078469, FJ183371, HM035361, HM035392, AF545433, M87876, NC 007754, NC 007663, NC 006004, ACF22097, AY485667, AM498057, FJ437558, AY841352, GQ506542, GQ506502, AF172829, AF188660, X53740, AY493692, M63417, AJ277802, AF172826, AF172825, AF188674, AF188673, AF172831, EU839843, L11724, DQ465027, DQ465028, DQ465026.

BLAST analysis of sequences from KUW2010/02 consistently showed highest levels of sequence identity to homologous genome segments of other BTV isolates. Results of phylogenetic analyses using CLUSTAL X and MAFFT alignments, neighbour-joining and maximum likelihood tree construction, all located the genome segments of KUW2010/02 within the BTV serogroup/species, confirming the results of BLAST analyses (Figures 1, 2 and 3 – see below). The use of neighbour-joining (p distance) and maximum likelihood methods did not alter the clustering or phylogenetic relationships of any KUW2010/02 genome segment to a great extent.

#### Segment 1

Comparisons of Seg-1 from KUW2010/02 with other BTVs, showed that it is conserved at 3944 base pairs (bp), encoding the 1302 amino acid (aa) of VP1[Pol] (Table 1). The sequences of Seg-1/VP1[Pol] are also highly ‘conserved’, with overall nt/aa identity levels of 74.6%/86.0% to 75.8%/87.8% to other BTV isolates and a maximum of 67.5%/68.8% to members of other *Orbivirus* species (EHDV-1/NIG1967/01 and EHDV-6/AUS1981/07, respectively), confirming its identification as a novel BTV isolate. However, KUW2010/02 does not cluster within either of the major BTV topotypes previously identified, showing similarly low maximum nt/aa identity levels to members of both ‘eastern’ and ‘western’ topotypes (Table 1) and to BTV-25 (SWI2008/01), which represents a distinct (western) topotype [16]. These data indicate that BTV-26 (KUW2010/02) also represents a further distinct (eastern) group/topotype.

#### Segment 2

Seg-2 of KUW2010/02 is 2929 bp long, encoding 957 aa of VP2 (Table 1) showing nt/aa identity levels of 42.8%/28.3% to 63.9%/61.5% to previously recognised BTV serotypes. As previously reported [39] these low values identify KUW2010/02 as a novel 26^th^ type within a distinct 12^th^ Seg-2 nucleotype ‘L’ [15], [16], [39]. The sizes of Seg-2 and VP2 of KUW2010/02 show differences in length when compared to other BTV serotypes. Seg-2 of KUW2010/02 showed slightly higher levels of identity to the SWI2008/01 strain of BTV-25, than to reference strains of BTV-10 and BTV-17 from the USA (Table 1).

#### Segment 3

Seg-3 of KUW2010/02 is 2773 bp long, encoding 901 aa of the highly conserved BTV sub-core-shell protein, VP3(T2) (Table 1), showing 73.7%/87.6% to 76.6%/88.9% nt/aa identity with other BTVs. Although lengths are otherwise conserved, the 3′ NCR of KUW2010/02 Seg-3 is one nucleotide longer than other BTV isolates that have been analysed (N = >80). Closest relationships were detected with ‘eastern’ strains of BTV-16 from Israel, and ‘western’ reference strains of BTV-2 and 9 (Table 1). Similar levels of identity were also detected with BTV-25 (SWI2008/01).

In comparisons with the T2 gene of multiple other *Orbivirus* species, KUW2010/02 showed a maximum of 69.9%/77.5% nt/aa identity with EHDV (EHDV-4/NIG1968/01). From previous studies these identity levels confirm KUW2010/02 as an isolate of BTV [16], [23]. None of the previously characterised BTV strains show much closer relationships to KUW2010/02 in Seg-3 (Figure 1, Table 1 and 2), indicating that it does not cluster within the previously recognised topotypes [16], [17], [21] and therefore (as indicated for Seg-1) represents a further distinct (eastern) group/topotype.

**Table 2:** Summary of percentage sequence identities for Seg-3/VP3[T2] between the eastern viruses, western viruses, BTV-25/SWI2008/01 and KUW2010/02.

	Major eastern topotype		Major western topotype		BTV-25 (SWI2008/01)		BTV-26(KUW2010/02)	
**Major eastern topotype**	>89.8	***>98.1***						
**Major western topotype**	79.3–82.4	***96.9*** **–** ***99.3***	>87.5	***>97.7***				
**BTV-25 (SWI2008/01)**	74.9–76.7	***88.0*** **–** ***88.8***	75.0–76.1	***88.5*** **–** ***89.5***	ID	***ID***		
**BTV-26 (KUW2010/02)**	74.6–75.8	***87.9*** **–** ***88.9***	75.0–76.4	***87.6*** **–** ***88.6***	76.6	***88.9***	ID	***ID***

Both nucleotide (nt) and amino acid (aa-bold italics) identities are presented.

#### Segment 4

Seg-4 of BTV is 1982 nt in length, encoding 644 aa of the highly conserved VP4 capping enzyme protein (CaP) (Table 1), showing nt/aa identity levels of 72.3%/79.3% to 74.8%/82.3% with other BTVs. Although lengths are otherwise conserved, the 3′ NCR of KUW2010/02 Seg-4 is one nucleotide longer than the other BTV isolates analysed (N = >70). Highest overall identity levels were detected between KUW2010/02 and BTV-10 USA (western topotype), BTV-16 Greece (eastern topotype) and to BTV-25 (SWI2008/01), consistent with membership of a distinct (eastern) group/topotype (Table 1).

#### Segment 5

Seg-5 of BTV-26 KUW2010/02 is 1758 nt long, encoding 552 aa of the highly conserved NS1 tubule protein (TuP). However, these lengths showed considerable variation, particularly in the 3′ NCR (Table 1), when compared to other BTV isolates (N = >85). Seg-5/NS1[TuP] of KUW2010/02 shows nt/aa identity levels of 72.5%/79.3% to 74.4%/81.2% with other BTVs, and closest relationships to BTV-9 from Serbia and Bulgaria (eastern toptotype) and BTV-8 from Nigeria and BTV-17 from Trinidad and Tobago (western topotype). Similar nt/aa identity levels were also detected with BTV-20 Australia (Ac. No. X56735) and BTV-25 (SWI2008/01), which individually form additional ‘far eastern’ and ‘western’ topotypes, again indicating that KUW2010/02 represents another discrete eastern group/topotype.

#### Segment 6

Seg-6 of BTV-26 KUW2010/02 is 1629 nt long, encoding the 523 aa of VP5, the smaller of the two outer-capsid components and second most variable of the BTV proteins (Table 1). This is the smallest Seg-6/VP5 that has been recorded for any BTV (by 9 nucleotides and 3 amino acids), showing nt/aa identity levels of only 57.1%/41.4% to 73.0%/79.3% to previously recognised BTV serotypes. Closest relationships were detected with BTV-21 Australia, BTV-9 Serbia (eastern topotype), reference strains of BTV-11, BTV-24 (western toptotype) and with BTV-25 (SWI2008/01) (Table 1). As seen for Seg-2/VP2, these identity levels also support the identification of KUW2010/02 as a distinct 26^th^ virus ‘type’, within a novel 9^th^ Seg-6 nucleotype ‘I’ [16] (Figure 2).

#### Segment 7

Seg-7 of KUW2010/02 is 1157 bp long, encoding 349 aa of the major BTV serogroup-specific antigen and core surface protein - VP7 (Table 1). These lengths are similar to those of some other but not all previously characterised BTV isolates (N = >100). Sequence comparisons of Seg-7/VP7[T13] confirmed KUW2010/02 as an isolate of BTV, with identity levels ranging from 69.2%/80.8% to 81.2%/97.7% to other isolates, and closest relationships with BTV-25 (Figure 3). Close relationships were also detected with BTV-23 from India and BTV-2 from China (eastern topotype); BTV-1 from France and BTV-5 from the USA (western topotype) (Table 1).

#### Segment 8

Seg-8 of KUW2010/02 is 1121 bp long encoding the 353 aa of the viral inclusion body (VIB) matrix protein - NS2 (Table 1). This Seg-8/NS2 is four nucleotides and one aa shorter than any BTV strain previously analysed (N = >98). Seg-8/NS2[ViP] of KUW2010/02 show nt/aa identity levels of 67.6%/65.9% to 71.6%/70.9% with other BTVs, and closest relationships to BTV-1 Australia, BTV-12 Taiwan (eastern topotype); reference strain of BTV-1 and BTV-17 from Trinidad and Tobago (western topotype) and BTV-25/TOV (Table 1). As observed with the other conserved segments, KUW2010/02 represents a second distinct ‘eastern’ topotype.

#### Segment 9

Seg-9 of the KUW2010/02 is 1070 bp, encodes VP6, a minor core protein and the helicase enzyme (Hel) of 336 aa in length, as well as NS4 (from an out of frame ORF), which is 77 aa in length [27] (Table 1). This is 18 nt/6 aa longer than Seg-9/VP6 of ‘eastern’ BTV strains (N = 51) and 21 nt/7 aa longer than Seg-9/VP6 of ‘western’ strains previously characterised (N = 102). Seg-9/VP6[Hel] from KUW2010/02, shows identity levels that range from 64.3%/53.6% to 73.7%/75.0% to other BTV isolates, with closest relationships to BTV-9 from Bosnia (eastern topotype), BTV-10 USA, the reference strain of BTV-3 (western topotype) and BTV-25 (Table 1). As with the other genome segments analysed, these data indicate that BTV-26 KUW2010/02 represents a further distinct ‘eastern’ topotype. As with other BTVs, NS4 of KUW2010/02 is also highly conserved.

**Figure 4:**
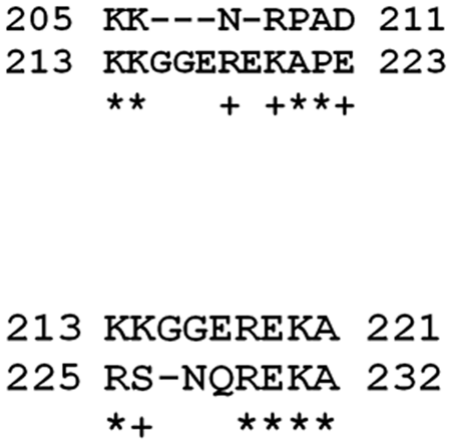
Examples of contiguous repeats found in the aa sequence of KUW2010/02 VP6. Evidence was detected for repeated contiguous aa sequences in VP6 of KUW2010/02. The aa positions, as indicated, are between residues 205 to 232. The region 213 to 223 is shown as the target sequence, with matching repeats 205–211 (upstream) and 225–232 (downstream), shown in the upper and lower lines respectively. + similar residue: * identical residue.

Three consecutive amino acid sequence repeats were identified within VP6 of KUW2010/02. These repeats which are shown in Figure 4, are located between codon positions 205 – 232 and may explain why VP6 of KUW2010/02 is so long. These repeats are outside the NS4 region (nt 185 – 415). Interestingly each repeat was found to align best with the protein sequence immediately upstream of it within in VP6. However, the repeated sequences are not fully identical. This suggests sequence duplication has been followed by some ‘evolution’ of the parental and the daughter repeated sequences [56], [57].

#### Segment 10

Seg-10 of KUW2010/02 is 822 bp long and codes for two, related non-structural proteins, NS3 (229 aa) and NS3a (216 aa) (Table 1). These lengths are identical to other BTV strains analysed (N = >95). Seg-10/NS3 of KUW2010/02 show nt/aa identity levels of 76.5%/84.3% to 82.6%/89.5% with other BTVs, and closest relationships with BTV-2 Taiwan and BTV-9 Greece (eastern strains), BTV-6 South Africa and BTV-8 Netherlands (western strains), and BTV-25 (SWI2008/01) (Table 1). In a pattern similar to other segments these data for Seg-10 of BTV-26 KUW2010/02 indicate that it represents the first isolate of a distinct ‘eastern’ topotype.

### Positive/negative selection analysis

The Tajima D test of neutrality, implemented in MEGA5, was used to assess selection. The expected value for populations that conform to a standard neutral model for selection is zero [57]. However the D values obtained for Seg-1 to Seg-10 reject the ‘null hypothesis’ for neutral selection of the BTV segments.

Recombination can adversely affect the power and accuracy of phylogenetic reconstruction [58] and may result in higher rates of false positives in maximum likelihood tests for positive selection [59].

No evidence of recombination was detected in Seg-4 to Seg-10 using GARD and RDP, whereas in Seg-1, Seg-2 and Seg-3 both programs showed evidence of one breakpoint, although the results were inconclusive. Positive selection analysis was performed separately for each genome segment of BTV. The SLAC and FEL methods did not identify any sites in the majority of the BTV genome segments with evidence of significant positive selection at the p, 0.1 level. However, in the Seg-9, 18 codon sites (5, 38, 63, 70, 72, 87, 90, 92, 93, 97, 98, 112, 119, 125, 126, 128, 131 and 132) (p = 0.09) were identified by the FEL method as being influenced by positive selection, where as SLAC only identified 6 codon sites (72, 87, 97, 125, 126 and 131) that were positively selected (p = 0.079). The majority of these positively selected codons are in the NS4 ORF in Seg-9 (between codons 60–138) [27].

Positive selection analyses were also performed separately for the eastern and western lineages of Seg-9. Seg-9 sequences of KUW2010/02 and SWI2008/01 were used in both eastern as well as western analysis, as each of them makes a separate eastern and western cluster respectively. The SLAC method did not identify any site in the eastern lineage which gave evidence of positive selection significant at the p 0.1 level. However, codons 75 and 97 in the western lineage, were identified by the SLAC method as influenced by positive selection (p = 0.65). FEL methods identified 6 (codon 5, 63, 64, 69, 70, 103) and 15 (5, 55, 63, 72, 75, 87, 92, 97, 98, 112, 119, 123, 125, 128, 131) positively selected sites in the eastern and western lineage respectively significant at the p 0.1 level.

The global estimate of dN/dS by SLAC method for Seg-1 to Seg-10 were 0.036, 0.132, 0.018, 0.0516, 0.051, 0.048, 0.022, 0.087, 0.242, 0.047, respectively (using estimated (default) option where dN/dS is estimated from the data), indicating purifying or strong purifying selection. A high number of negatively selected codons were also identified (significant at the p = 0.1 level) in each genome segment with SLAC and FEL (data not shown) suggesting that all BTV genes evolved under negative/purifying selection.

### RT-PCR assays

**Figure 5:**
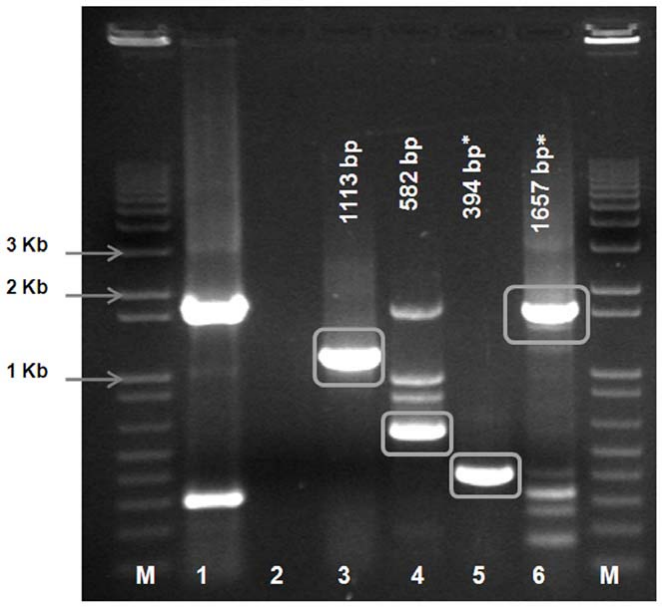
Electrophoretic analysis of cDNA products generated from Seg-2 of BTV-26 (KUW2010/02) using primer-pairs designed from the homologous sequence. PCR amplicons were generated from Seg-2 of BTV-26, isolate KUW2010/02 using primer- pairs 1 – 4 - Table 3 (lanes 3 to 6 respectively). Primer-pairs 3 and 4 are BTV-26 specific, while primer-pairs 1 and 2 also amplifies certain other serotypes in Seg-2 nucleotype ‘A’. Lane 1 is a positive control using RNA from BTV-6/RSArrrr/06, with primer-pair BTV-6/2/301F & BTV-6/2/790R – 1631 bp [16]. Lane 2 is a negative water control. Lane M: 1 kb marker.

Sequence data generated for Seg-2 of KUW2010/02, and comparisons to other BTV types, were used to design four sets of oligonucleotide primers for conventional RT-PCR assays (Table 3). All four primer-pairs (1 to 4) worked well, generating products of the expected sizes from the original blood sample (KUW2010/01) and both passage levels of BTV-26 (KUW2010/02 and KU2010/03) (Figure 5). Although other combinations of these forward and reverse primers also appeared to be effective, they were not widely evaluated (data not shown). Primer-pairs 1 to 4 were also tested with reference strains of the most closely related heterologous serotypes (BTV-4, 10, 11, 17, 20 and 24, belonging to nucleotype ‘A’ [15]. Primer-pairs 1 and 2 generated faint bands that were near to the ‘predicted’ size, with some strains from nucleotype ‘A’ (Primer-pair 1 with BTV-4, 10, 17, 20 and 24; Primer-pair 2 with BTV-4, 10 and 17). Therefore primer-pairs 1 and 2 are not considered to be entirely BTV-26 specific. However, any cDNA amplicons generated can be sequenced using the same primer sets, helping to identify both the virus strain and its relationships to other isolates.

**Table 3:** Primers for amplification of Seg-2 from BTV-26 in RT-PCR assays.

Primer Pair	Primer Name*	Primer Sequence (5′-3′)	Position on genome Seg-2 (nt)	Predicted Product size (bp)
**Pair 1**	BTV-26/S2/176-196F BTV-26/S2/1289-1268R	TCTAAGCAAGGGATTATCGAT TAACTTCCTCATCAACTGAGAT	176–196 1289–1268	1113†
**Pair 2**	BTV-26/S2/1267-1286F BTV-26/S2/1849-1831R	TATCTCAGTTGATGAGGAAG GCATATATCCCTTTCACCT	1267–1286 1849–1831	582†
**Pair 3**	BTV-26/S2/1819-1839F BTV-26/S2/2213-2194R	ACATTACGCTAGAGGTGAAAG GATCACGAATCACCTCGACG	1819–1839 2213–2194	394‡
**Pair 4**	BTV-26/S2/286-303F BTV-26/S2/1943-1919R	GATGAGGACAGCACGGAA GACCGTGGTGATATTGTGGATCAAG	286–303 1943–1919	1657‡

*Individual primers are identified by the BTV serotype (e.g. BTV-26) followed by the letter S and number 2 (to indicate Seg-2), then a number to indicate the relative nucleotide position of the primer within VP2 gene, followed by F or R to indicate forward or reverse orientation.

† Primer-pairs 1 and 2 also generated very faint but near right sized bands from Seg-2 of certain serotypes within nucleotype ‘A’ (BTV-4, 10, 17, 20 and 24 – Primer-pair 1; BTV-4, 10 and 17 – Primer-pair 2), the most closely related nucleotype/serotypes to BTV-26 and therefore they cannot be regarded as BTV-26 specific.

‡ Primer-pairs 3 and 4 although generated multiple bands of low intensity with some serotypes in the nucleotype ‘A’ but none of them was of right size, so these two sets can be regarded as BTV-26 specific.

Although primer-pairs 3 and 4 generated multiple low intensity bands with RNA from some of the serotypes in the nucleotype ‘A’, none of these products were the correct size, and these two sets are therefore regarded as BTV-26 specific. In each case unambiguous identification of BTV- 26 can also be achieved by sequencing and phylogenetic comparisons to the cDNA generated (as described here). KUW2010/02 represents a reference stain for the novel BTV serotype 26.

## Discussion

Many viruses with RNA genomes can rapidly adapt to and exploit rapidly changing global landscapes and local environments. Genetic variation (mutation, recombination, and reassortment) and environmental factors (including trade, ecosystem, communal, and health care factors) can play important roles in the selection, emergence and evolution of different viruses. This paper presents full genome sequence data for the reference strain of a novel BTV serotype (BTV-26) for further comparative studies.

Blood/tissue and serum samples, from sheep and goats in Kuwait showing clinical signs of disease (suspected BTV infection), were sent from the Diagnostic Laboratory Centre (PAAF-Kuwait) to IAH-UK for testing. Most of the serum samples were positive for BTV specific antibodies, indicating previous BTV infection (there is no BTV vaccination policy in Kuwait). However, BTV-RNA was only detected in two sheep blood samples (animals 364 and 374) using a BTV-Seg-9 (Maan et al – in preparation) and BTV-Seg-10 specific rRT-PCR assay (designed by Orru et al [37] that had previously also been used to detect BTV-25 in Switzerland [14], suggesting that the ongoing and more widespread clinical signs observed were not due to a current BTV infection. However, BTV Seg-1, or Seg-1 and 5 specific assays [53], [54] failed to detect RNA of the Kuwait virus, indicating that it was an unusual or atypical BTV strain. Experimental infections of sheep with KUW2010/02 caused only mild clinical disease (Chris Oura – Personal communication). Further diagnostic, pathogenesis and insect transmission studies will add to our knowledge of this novel BTV serotype/topotype.

### Identification of KUW2010/02 as an isolate of BTV

When analysed by AGE, KUW2010/02 generated a migration pattern typical of a BTV isolate, indicating that it is a member of this virus species [39].

Earlier studies of Seg-3/VP2[T2] from different orbiviruses, initially showed >91% aa identity within the same species/serogroup [24]. However, subsequent studies that included multiple BTV isolates from different geographic regions (topotypes), detected as little as 74.9% nt/87.8% aa identity in Seg-3/VP3 [16].

In the study presented here, Seg-3/VP3 of KUW2010/02 showed up to 76.6% nt/88.9% aa identity with other BTV strains (Table 1), confirming that it belongs to the same virus species. However, 73.7% nt identity with BTV-15 Australia [Ac. No. AY322427] and 87.6% aa identity with BTV-2 USA [Ac. No. L19967], have further reduced the lower identity limits detected within the species. Similar results were obtained with the other conserved genome segments (Seg-1, -4, -5, -7, -8, -9 and -10), in each case confirming KUW2010/02 as an isolate of BTV, although again slightly reducing the lower limit of identity detected between BTV isolates in Seg-1, -4, -8 and -9.

VP7[T13] is the major serogroup-specific antigen of BTV and related orbiviruses [22], [60]. KUW2010/02 not only gave high-level positive results in a BTV-specific antigen-ELISA targeting VP7 [41], it also showed up to 97.7% nt/aa identity to another BTV strain (SWI2008/01), consistent with its identity as a member of the *Bluetongue virus* species.

### Identification of KUW2010/02 as BTV-26

Neutralisation assays demonstrated that none of the antisera against BTV-1 to BTV-25, caused significant levels of neutralisation, indicating that KUW2010/02 belongs to a novel 26^th^ BTV type [39].

Seg-2/VP2 of KUW2010/02 showed a maximum of 63.9% nt and 61.5%/aa identity with BTV-25 (SWI2008/01). These levels are significantly lower than previously detected within a single BTV serotype (minimum levels of 68.4% nt/72.6% aa – [16]), confirming the identification of KUW2010/02 as BTV-26, and as a 12^th^ Seg-2 nucleotype (L). However, these values also slightly increase the maximum level of identity detected between different BTV serotypes (previous maximum of 61.4% nt/59.5% aa).

We have designed two initial pairs of conventional primers for the amplification and detection of Seg-2 from KUW2010/02, which do not amplify Seg-2 of other BTV serotypes and in this respect can be regarded as ‘type specific’. However, we recognise that other strains of BTV-26 may be isolated in future, which have sequence differences in the footprints of these initial primer sets. Seg-2 of any such viruses will need to be sequenced, so that these ‘type-specific’ primers can be redesigned, maintaining their specificity.

Seg-6/VP5, which can also influence BTV serotype [13], showed a maximum of 73.0% nt/79.3% aa identity between KUW2010/02 and any other BTV type (Table 1), indicating that it belongs to a distinct and 9^th^ Seg-6 nucleotype (I) (Figure 2) [16], [17]. This is again consistent with its identification as BTV-26. The lowest similarity detected in Seg-6/VP5 between KUW2010/02 and other BTV serotypes was 57.1% nt and 41.4% aa, slightly above levels previously detected between BTV-25 strain SWI2008/01 and other BTV isolates (at 56.9% nt and 40.8% aa) [16].

We therefore propose KUW2010/02 as the reference strain for this novel serotype, with the Seg-2 specific primer-pairs designed for conventional RT-PCR assays and sequencing studies, providing initial diagnostic tools for BTV-26.

### Identification of KUW2010/02 as a novel major topotype

Most BTV isolates can be divided between two major ‘eastern’ or ‘western’ topotypes (reflecting their geographic origins) then into a number of further geographic subgroups based on phylogenetic analyses of their genome segments [16], [17]. Viruses within the same major-topotype showed >87.5% nt identity in Seg-3, while a maximum of 82.4% nt identity was detected between the major eastern and western groups/topotypes (Table 2). The data presented here show a maximum of 75.8%, 76.4% or 76.6% nt identity between Seg-3 of KUW2010/02 and eastern topotype, western topotype or BTV-25 respectively. These data indicate that KUW2010/02 and BTV-25 (SWI2008/01) represent two new and distinct groups of Seg-3 sequences [16], and may therefore represent additional ‘major’ topotypes (Figure 1, Table 1 and 2).

### Evolutionary selection of BTV sequences

All of the BTV genes, including those coding for VP2 – VP7 and NS1 - NS3, appear to have evolved under purifying selection (sometimes strongly so), evidenced by the dN/dS values of <1. Relatively high dN/dS value suggested of that protein translated from Seg-2 and Seg-9 might be targets for periodic positive selection. The majority of positively selected codons in Seg-9, fall in the ORF for NS4 (60–138 aa) [27], indicating significant functional constraints. Importantly, VP2 determines BTV serotype and is the most variable segment in the viral genome, whereas Seg-9 encodes the viral helicase VP6 and NS4, which is highly conserved in all BTVs. The role of NS4 has yet to be identified, although bioinformatic analyses indicate that it contains coiled-coils and is related to proteins that bind nucleic acids, or are associated with lipids or membranes. The results obtained for Seg-2, 3, 6 and 10 are consistent with previous conclusions [21], [61], [62]. Lee et al. [63] have also reported similar findings, except for the VP7 gene, which they suggest has a positive or diversifying selection (dN/dS ratios ranged from 1.2 to 5.7 (2.85±2.0; n = 4)). In contrast we find using greater numbers of VP7 sequences and a more diverse data set, that negative or purifying selection dominates the evolution of all BTV genes, most likely due to the constraint imposed by the alternate arthropod-vertebrate host transmission cycle. There are reports that some other vector-borne RNA viruses including West Nile virus [64] and Venezuela equine encephalitis virus [65] also evolve under purifying selection [66].

Sequence comparisons of most of the conserved genes and proteins place KUW2010/02 and SWI2008/01 (BTV-25) in additional but distinct geographic groups (representing additional major topotypes of BTV). KUW2010/02 and SWI2008/01 show only 81.2% nt sequence identity in Seg-7, again indicating that they have evolved separately as members of distinct geographic groups (topotypes) a for long period of time. However, a very high level of aa identity (97.7%) was detected in VP7[T13] between KUW2010/02 and SWI2008/01, indicating that they share a common ancestry and suggesting very strong conservation pressures / functional constraints on the sequence of VP7 between these two strains. It is therefore possible for conservation pressures on aa sequence to mask the regional variations between orbivirus topotypes, even though they are still evident as relatively large variations in nt sequence.

The provision of a full genome sequence for the novel BTV serotype (BTV-26) will make it possible to track any further changes, or reassortment events, that occur if BTV-26 continues to persist or spread in the region.

## References

[1] Mertens PPC, Maan S, Samuel A, Attoui H, Fauquet CM, Mayo M.A., Maniloff J., Desselberger U., Ball L.A. (2005). Orbiviruses, Reoviridae.. Virus Taxonomy Eighth Report of the International Committee on Taxonomy of Viruses.

[2] Attoui H, Maan SS, Anthony SJ, Mertens PPC (2009). Bluetongue virus, other orbiviruses and other reoviruses: Their relationships and taxonomy..

[3] Alexander KA, MacLachlan NJ, Kat PW, House C, O'Brien SJ (1994). Evidence of natural bluetongue virus infection among African carnivores.. Am J Trop Med Hyg.

[4] Ruiz-Fons F, Reyes-Garcia AR, Alcaide V, Gortazar C (2008). Spatial and temporal evolution of bluetongue virus in wild ruminants, Spain.. Emerg Infect Dis.

[5] Meyer G, Lacroux C, Leger S, Top S, Goyeau K (2009). Lethal bluetongue virus serotype 1 infection in llamas.. Emerg Infect Dis.

[6] Wilson AJ, Mellor PS (2009). Bluetongue in Europe: past, present and future.. Philos Trans R Soc Lond B Biol Sci.

[7] Maclachlan NJ, Guthrie AJ (2010). Re-emergence of bluetongue, African horse sickness, and other orbivirus diseases.. Vet Res.

[8] Howerth EW, Greene CE, Prestwood AK (1988). Experimentally induced bluetongue virus infection in white-tailed deer: coagulation, clinical pathologic, and gross pathologic changes.. Am J Vet Res.

[9] Darpel KE, Batten CA, Veronesi E, Shaw AE, Anthony S (2007). Clinical signs and pathology shown by British sheep and cattle infected with bluetongue virus serotype 8 derived from the 2006 outbreak in northern Europe.. Vet Rec.

[10] MacLachlan NJ (1994). The pathogenesis and immunology of bluetongue virus infection of ruminants.. Comp Immunol Microbiol Infect Dis.

[11] Mertens PP, Brown F, Sangar DV (1984). Assignment of the genome segments of bluetongue virus type 1 to the proteins which they encode.. Virology.

[12] Roy P (1989). Bluetongue virus genetics and genome structure.. Virus Res.

[13] Mertens PP, Pedley S, Cowley J, Burroughs JN, Corteyn AH (1989). Analysis of the roles of bluetongue virus outer capsid proteins VP2 and VP5 in determination of virus serotype.. Virology.

[14] Hofmann MA, Renzullo S, Mader M, Chaignat V, Worwa G (2008). Genetic characterization of toggenburg orbivirus, a new bluetongue virus, from goats, Switzerland.. Emerg Infect Dis.

[15] Maan S, Maan NS, Samuel AR, Rao S, Attoui H (2007). Analysis and phylogenetic comparisons of full-length VP2 genes of the 24 bluetongue virus serotypes.. J Gen Virol.

[16] Maan S, Maan NS, van Rijn PA, van Gennip RG, Sanders A (2010). Full genome characterisation of bluetongue virus serotype 6 from the Netherlands 2008 and comparison to other field and vaccine strains.. PLoS One.

[17] Maan S, Maan NS, Ross-smith N, Batten CA, Shaw AE (2008). Sequence analysis of bluetongue virus serotype 8 from the Netherlands 2006 and comparison to other European strains.. Virology.

[18] Huismans H, Erasmus BJ (1981). Identification of the serotype-specific and group-specific antigens of bluetongue virus.. Onderstepoort J Vet Res.

[19] Roy P (2008). Functional mapping of bluetongue virus proteins and their interactions with host proteins during virus replication.. Cell Biochem Biophys.

[20] Pritchard LI, Gould AR, Wilson WC, Thompson L, Mertens PP (1995). Complete nucleotide sequence of RNA segment 3 of bluetongue virus serotype 2 (Ona-A). Phylogenetic analyses reveal the probable origin and relationship with other orbiviruses.. Virus Res.

[21] Nomikou K, Dovas CI, Maan S, Anthony SJ, Samuel AR (2009). Evolution and phylogenetic analysis of full-length VP3 genes of Eastern Mediterranean bluetongue virus isolates.. PLoS One.

[22] Gumm ID, Newman JF (1982). The preparation of purified bluetongue virus group antigen for use as a diagnostic reagent.. Arch Virol.

[23] Attoui H, Mohd Jaafar F, Belhouchet M, Aldrovandi N, Tao S (2005). Yunnan orbivirus, a new orbivirus species isolated from Culex tritaeniorhynchus mosquitoes in China.. J Gen Virol.

[24] Attoui H, Mendez-Lopez MR, Rao S, Hurtado-Alendes A, Lizaraso-Caparo F (2009). Peruvian horse sickness virus and Yunnan orbivirus, isolated from vertebrates and mosquitoes in Peru and Australia.. Virology.

[25] Grimes JM, Burroughs JN, Gouet P, Diprose JM, Malby R (1998). The atomic structure of the bluetongue virus core.. Nature.

[26] Roy P (1992). Bluetongue virus proteins.. J Gen Virol.

[27] Belhouchet M, Mohd Jaafar F, Tesh R, Grimes J, Maan S (2010). Complete sequence of Great Island virus and comparison with the T2 and outer-capsid proteins of Kemerovo, Lipovnik and Tribec viruses (genus Orbivirus, family Reoviridae).. J Gen Virol.

[28] Firth AE (2008). Bioinformatic analysis suggests that the Orbivirus VP6 cistron encodes an overlapping gene.. Virol J.

[29] Huismans H, Els HJ (1979). Characterization of the tubules associated with the replication of three different orbiviruses.. Virology.

[30] Huismans H, van Dijk AA, Bauskin AR (1987). In vitro phosphorylation and purification of a nonstructural protein of bluetongue virus with affinity for single-stranded RNA.. J Virol.

[31] Huismans H, van Staden V, Fick WC, van Niekerk M, Meiring TL (2004). A comparison of different orbivirus proteins that could affect virulence and pathogenesis.. Vet Ital.

[32] Quan M, van Vuuren M, Howell PG, Groenewald D, Guthrie AJ (2008). Molecular epidemiology of the African horse sickness virus S10 gene.. J Gen Virol.

[33] Gould AR (1987). The complete nucleotide sequence of bluetongue virus serotype 1 RNA3 and a comparison with other geographic serotypes from Australia, South Africa and the United States of America, and with other orbivirus isolates.. Virus Res.

[34] Purse BV, Mellor PS, Rogers DJ, Samuel AR, Mertens PP (2005). Climate change and the recent emergence of bluetongue in Europe.. Nat Rev Microbiol.

[35] Purse BV, Brown HE, Harrup L, Mertens PP, Rogers DJ (2008). Invasion of bluetongue and other orbivirus infections into Europe: the role of biological and climatic processes.. Rev Sci Tech.

[36] Johnson DJ, Mertens PPC, Maan S, Ostlund EN (2007). In Proceedings of Annual Conference of American Association of Veterinary Laboratory Diagnosticians (AAVLD).. Proceedings of Annual Conference of American Association of Veterinary Laboratory Diagnosticians (AAVLD).

[37] Orru G, Ferrando ML, Meloni M, Liciardi M, Savini G (2006). Rapid detection and quantitation of Bluetongue virus (BTV) using a Molecular Beacon fluorescent probe assay.. J Virol Methods.

[38] Planzer J, Kaufmann C, Worwa G, Gavier-Widen D, Hofmann MA (2011). In vivo and in vitro propagation and transmission of Toggenburg orbivirus.. Res Vet Sci.

[39] Maan S, Maan NS, Nomikou K, Batten C, Antony F (2011). Novel bluetongue virus serotype from Kuwait.. Emerg Infect Dis.

[40] Mertens PPC, Attoui H (2011a). The RNAs and proteins of dsRNA viruses.. http://www.reoviridae.org/dsRNA_virus_proteins/ReoID/viruses-at-iah.htm.

[41] Thevasagayam JA, Wellby MP, Mertens PP, Burroughs JN, Anderson J (1996). Detection and differentiation of epizootic haemorrhagic disease of deer and bluetongue viruses by serogroup-specific sandwich ELISA.. J Virol Methods.

[42] Grist NR, Ross CA, Bell EJ (1974). 2nd ed..

[43] Attoui H, Billoir F, Cantaloube JF, Biagini P, de Micco P (2000). Strategies for the sequence determination of viral dsRNA genomes.. J Virol Methods.

[44] Maan S, Rao S, Maan NS, Anthony SJ, Attoui H (2007). Rapid cDNA synthesis and sequencing techniques for the genetic study of bluetongue and other dsRNA viruses.. J Virol Methods.

[45] Thompson JD, Gibson TJ, Plewniak F, Jeanmougin F, Higgins DG (1997). The CLUSTAL_X windows interface: flexible strategies for multiple sequence alignment aided by quality analysis tools.. Nucleic Acids Res.

[46] Katoh K, Asimenos G, Toh H (2009). Multiple alignment of DNA sequences with MAFFT.. Methods Mol Biol.

[47] Wernersson R, Pedersen AG (2003). RevTrans: Multiple alignment of coding DNA from aligned amino acid sequences.. Nucleic Acids Res.

[48] Tamura K, Peterson D, Peterson N, Stecher G, Nei M (2011). MEGA5: Molecular Evolutionary Genetics Analysis using Maximum Likelihood, Evolutionary Distance, and Maximum Parsimony Methods.. Mol Biol Evol.

[49] Delport W, Poon AF, Frost SD, Kosakovsky Pond SL (2010). Datamonkey 2010: a suite of phylogenetic analysis tools for evolutionary biology.. Bioinformatics.

[50] Martin DP, Lemey P, Lott M, Moulton V, Posada D (2010). RDP3: a flexible and fast computer program for analyzing recombination.. Bioinformatics.

[51] Suzuki Y, Gojobori T (1999). A method for detecting positive selection at single amino acid sites.. Mol Biol Evol.

[52] Mertens PP, Maan NS, Prasad G, Samuel AR, Shaw AE (2007). Design of primers and use of RT-PCR assays for typing European bluetongue virus isolates: differentiation of field and vaccine strains.. J Gen Virol.

[53] Shaw AE, Monaghan P, Alpar HO, Anthony S, Darpel KE (2007). Development and initial evaluation of a real-time RT-PCR assay to detect bluetongue virus genome segment 1.. J Virol Methods.

[54] Toussaint JF, Sailleau C, Breard E, Zientara S, De Clercq K (2007). Bluetongue virus detection by two real-time RT-qPCRs targeting two different genomic segments.. J Virol Methods.

[55] Mertens PPC, Attoui H (2011b). The RNAs and proteins of dsRNA viruses.. http://www.reoviridae.org/dsRNA_virus_proteins/CPV-RNA-Termin.htm.

[56] Gibbs A, Keese PK (1995). In search of the origins of viral genes..

[57] Attoui H, Jaafar FM, Belhouchet M, de Micco P, de Lamballerie X (2006). Micromonas pusilla reovirus: a new member of the family Reoviridae assigned to a novel proposed genus (Mimoreovirus).. J Gen Virol.

[58] Posada D, Crandall KA (2002). The effect of recombination on the accuracy of phylogeny estimation.. J Mol Evol.

[59] Anisimova M, Bielawski JP, Yang Z (2001). Accuracy and power of the likelihood ratio test in detecting adaptive molecular evolution.. Mol Biol Evol.

[60] Anthony S, Jones H, Darpel KE, Elliott H, Maan S (2007). A duplex RT-PCR assay for detection of genome segment 7 (VP7 gene) from 24 BTV serotypes.. J Virol Methods.

[61] Balasuriya UB, Nadler SA, Wilson WC, Pritchard LI, Smythe AB (2008). The NS3 proteins of global strains of bluetongue virus evolve into regional topotypes through negative (purifying) selection.. Vet Microbiol.

[62] Carpi G, Holmes EC, Kitchen A (2010). The evolutionary dynamics of bluetongue virus.. J Mol Evol.

[63] Lee F, Ting LJ, Lee MS, Chang WM, Wang FI (2011). Genetic analysis of two Taiwanese bluetongue viruses.. Vet Microbiol.

[64] Jerzak G, Bernard KA, Kramer LD, Ebel GD (2005). Genetic variation in West Nile virus from naturally infected mosquitoes and birds suggests quasispecies structure and strong purifying selection.. J Gen Virol.

[65] Coffey LL, Vasilakis N, Brault AC, Powers AM, Tripet F (2008). Arbovirus evolution in vivo is constrained by host alternation.. Proc Natl Acad Sci U S A.

[66] Woelk CH, Holmes EC (2002). Reduced positive selection in vector-borne RNA viruses.. Mol Biol Evol.

